# Supplementary material for: Impact of Melatonin Deficit on Emotional Status and Oxidative Stress-Induced Changes in Sphingomyelin and Cholesterol Level in Young Adult, Mature, and Aged Rats
Source: Int J Mol Sci. 2022 Mar 4;23(5):2809. doi: 10.3390/ijms23052809 (PMC8911298; doi:10.3390/ijms23052809)
Supplement: Supplementary file 1 [file ijms-23-02809-s001.zip › ijms-1621021-supplementary.pdf]

International Journal of *Molecular Sciences*

MDPI

Article

Impact of Melatonin Deficit on Emotional Status and Oxidative Stress-Induced Changes in Sphingomyelin and Cholesterol Level in Young Adult, Mature, and Aged Rats

Jana Tchekalarova [1,*], Zlatina Nenchovska [1], Lidia Kortenska [1], Veselina Uzunova [2], Irina Georgieva [2] and Rumiana Tzoneva [2]

[1] Institute of Neurobiology, Bulgarian Academy of Sciences, Acad. G. Bonchev Street, Block 23, 1113 Sofia, Bulgaria; zuzania@abv.bg (Z.N.); lkortenska@abv.bg (L.K.)
[2] Institute of Biophysics and Biomedical Engineering, Bulgarian Academy of Sciences, Acad. G. Bonchev Street, Block 21, 1113 Sofia, Bulgaria; georgieva.irina5@gmail.com (I.G.); vesi.uzunova@abv.bg (V.U.); tzoneva@bio21.bas.bg (R.T.)
* Correspondence: jt.chekalarova@inb.bas.bg

Table S1. Effects of factors Age, Surgery and Age x Surgery interaction on motor activity, anxiety and depressive-like behavior, measured in the open field (OF) test, sucrose preference test (SPT) and splash test. Two-way analysis of variance was performed followed by a *post hoc* Bonferroni when appropriate.

Behavioral tests	Two-way ANOVA
OF- Total distance	Age [$F_{2, 95}$ = 27,610; p < 0.001]<br>Surgery [$F_{2, 95}$ = 1,554; p =2.18]<br>Age x Surgery interaction [$F_{2, 95}$ = 1,918; p =0.156]
OF- Number of rearings	Age [$F_{2, 95}$ = 10,913; p < 0.001]<br>Surgery [$F_{2, 95}$ = 0,0551; p =0.815]<br>Age x Surgery [$F_{2, 95}$ = 4,411; p = 0.017]
OF- Distance center	Age [$F_{2, 95}$ = 21,921; p < 0.001]<br>Surgery [$F_{2, 95}$ = 2,899; p =0.095]<br>Age x Surgery interaction [$F_{2, 95}$ = 1,898; p =0.161]
OF- Time center	Age [$F_{2, 95}$ = 14,966; p < 0.001]<br>Surgery [$F_{2, 95}$ = 2,694; p =0.108]<br>Age x Surgery interaction [$F_{2, 95}$ = 1,626; p =0.208]
SPT	Age [$F_{2, 95}$ = 1.012; p = 0.372]<br>Surgery [$F_{2, 95}$ = 6.180; p =0.017]<br>Age x Surgery [$F_{2, 95}$ = 4.245; p = 0.021]
Splash test	Age [$F_{2, 95}$ = 1.283, p = 0.288]<br>Surgery [$F_{2, 95}$ = 13.971; p < 0.001]<br>Age x Surgery interaction [$F_{2, 95}$ = 0.172; p = 0.843]
ORT (number of counts)	Age [$F_{2, 95}$ = 0,411; p = 0.525]<br>Surgery [$F_{2, 95}$ = 4,314; p = 0.02]<br>Age x Surgery interaction [$F_{2, 95}$ = 0,902; p =0.414]
ORT (time)	Age [$F_{2, 95}$ = 14,966; p < 0.001]<br>Surgery [$F_{2, 95}$ = 2,694; p =0.108]<br>Age x Surgery interaction [$F_{2, 95}$ = 1,626; p =0.208]

Table S2. Effects of factors Age, Surgery and Age x Surgery interaction on oxidative stress markers, superoxide dismutase (SOD), glutathione (GSH), malondialdehyde (MDA). Two-way analysis of variance was performed followed by a *post hoc* Bonferroni when appropriate.

**Oxidative stress**	
SOD	Age [$F_{2, 95}$ = 5,468; p = 0.011]<br>Surgery [$F_{2, 95}$ = 6.135; p = 0.021]<br>Age x Surgery [$F_{2, 95}$ = 0.0337; p = 0.967]
GSH	Age [$F_{2, 95}$ = 1,009; p =0.382]<br>Surgery [$F_{2, 95}$= 5,198; p=0.038]<br>Age x Surgery [$F_{2, 95}$ = 0.194; p = 0.324]
MDA	Age [$F_{2, 95}$ = 31,534; p < 0.001]<br>Surgery [$F_{2, 95}$= 4,28; p = 0.0037]<br>Age x Surgery [$F_{2, 95}$ = 1.427; p = 0.248]
